# Supplementary material for: The Role of microRNAs Related to Apoptosis for N-Methyl-d-Aspartic Acid-Induced Neuronal Cell Death in the Murine Retina
Source: Int J Mol Sci. 2024 Jan 16;25(2):1106. doi: 10.3390/ijms25021106 (PMC10816001; doi:10.3390/ijms25021106)
Supplement: Supplementary file 1 [file ijms-25-01106-s001.zip › ijms-2822570-supplementary.pdf]

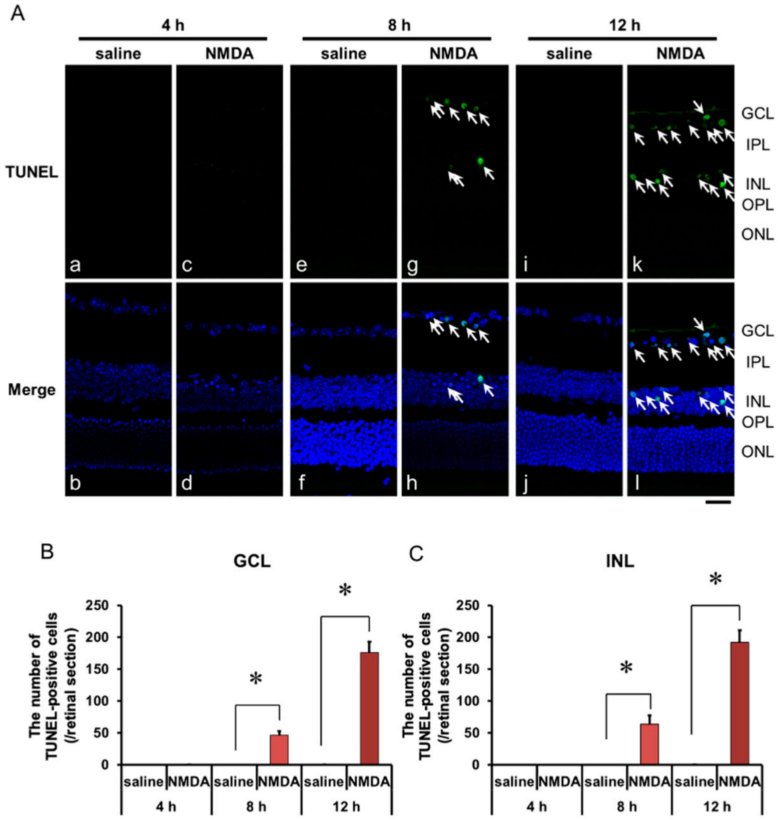

Figure S1. (A) Photomicrographs showing TUNEL staining of the retinas. The eyes were excised 4 **(a, b, c, d)**, 8 **(e, f, g, h)** and 12 **(i, j, k, l)** hours after intravitreal injection of saline (vehicle) or 40 nmol/eye NMDA. Panels b, d, f, h, i and l (Merge) show the TUNEL staining merged with nuclear counterstaining with DAPI. GCL: the ganglion cell layer, IPL: the inner plexiform layer, INL: the inner nuclear layer, OPL: the outer plexiform layer, and ONL: the outer nuclear layer. The nuclei of TUNEL-positive cells are stained green. All the nuclei are counterstained blue. The white arrows represent the TUNEL-positive cells. Scale bar is 50 μm. Original magnification, ×400. **(B, C)** The numbers of the TUNEL-positive cells in GCL **(B)** and INL **(C)** per one retinal section are shown. Each datum is presented as mean ± SEM of 3 to 5 independent experiments. *p<0.05, between the indicated pairs using Tukey–Kramer test.

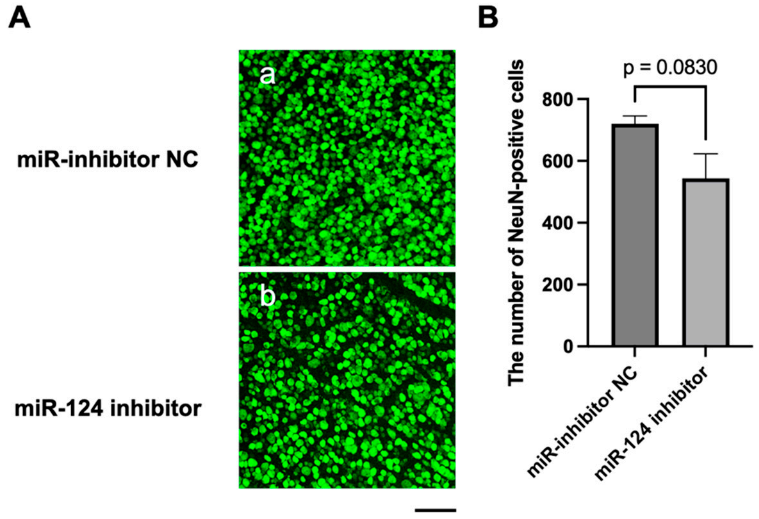

Figure S2. (A) The images of whole mount of the retina of the eyes treated with 10 nmol/eye miR-inhibitor NC **(a)** and 10 nmol/eye miR-124 inhibitor **(b)**. The eyes were excised 7 days and 18 hours after intravitreal injection of miR-inhibitor NC and miR-124 inhibitor. The flat-mount retina was stained with Alexa Fluor 488-conjugated anti-NeuN antibody to visualize the neurons in the ganglion cell layer (GCL). Scale bar is 100 μm. Original magnification, ×200. **(B)** The numbers of the NeuN-positive cells in GCL in an area of 0.2 mm^2 nearby the optic nerve head 7 days and 18 hours after intravitreal injection of miR-inhibitor NC and miR-124 inhibitor are shown. Each datum is presented as mean ± SEM of 5 to 6 independent experiments.
